# The Association between Stress and Children’s Weight Status: A School-Based, Epidemiological Study

**DOI:** 10.3390/children9071066

**Published:** 2022-07-17

**Authors:** Aikaterini Kanellopoulou, Christina Vassou, Ekaterina N. Kornilaki, Venetia Notara, George Antonogeorgos, Andrea Paola Rojas-Gil, Areti Lagiou, Mary Yannakoulia, Demosthenes B. Panagiotakos

**Affiliations:** 1 Department of Nutrition and Dietetics, School of Health Science and Education, Harokopio University, 17671 Athens, Greece; katerkane@gmail.com (A.K.); cvassou@hua.gr (C.V.); gantonogeorgos@gmail.com (G.A.); myianna@hua.gr (M.Y.); 2 Department of Preschool Education, School of Education, University of Crete, 74100 Rethimno, Greece; ekornilaki@edc.uoc.gr; 3 Laboratory of Hygiene and Epidemiology, Department of Public and Community Health, School of Public Health, University of West Attica, 12243 Athens, Greece; venotara@uniwa.gr (V.N.); alagiou@uniwa.gr (A.L.); 4 Department of Nursing, Faculty of Health Sciences, University of Peloponnese, 22100 Tripoli, Greece; apaola71@yahoo.com.mx; 5 Faculty of Health, University of Canberra, Canberra 2617, Australia

**Keywords:** child, obesity, overweight, schools, stress, public health

## Abstract

Given the evidence on how stress affects weight status in children, this research examined this association among Greek students aged 10–12 years old. Overall, 1452 children and their parents from several urban areas participated in an observational study conducted during the period 2014–2016. Participants completed validated questionnaires. International Obesity Task Force guidelines were used for children’s weight status classification. Descriptive statistics and nested logistic regression models were used. Multivariate correspondence analysis was also used to construct a score to evaluate the children’s stress levels. The overall prevalence of overweight/obesity was 27%. More than 80% of the children appeared to have a medium or high level of stress, mainly due to the school environment. School-related stress increased the odds of obesity in children. The association between stress and overweight/obesity status showed a consistent trend (adjusted odds ratios varied from 1.44 to 1.52, *p*-values < 0.01). Children’s weight status was associated with several school-related stressors. Although the school environment may play an aggravating role in the weight status of children, family plays a catalyst role in this direction. Therefore, actions have to be promoted in the school community so that children become more health literate on a public health level.

## 1. Introduction

In recent years, obesity has become a multifactorial and serious issue for public health [1]. Over the past five decades, obesity rates have rapidly increased by more than half worldwide and have affected both adults and children [2]. Greece is among the European countries with the highest rates of overweight and obesity in children [3]. In particular, in our country, the prevalence of overweight/obesity is 31.2% in boys and 29.1% in girls [3,4]. Factors considered serious for obesity comprise multifaceted genetics and biological, socioeconomic, environmental, or behavioral bases [5].

A major reason why obesity rates are rising rapidly in adults is the fact that people’s lifestyles have changed dramatically in recent decades. Today, people spend many hours sedentary and immobile, usually due to work, while meals with a high-calorie content are very easily consumed due to different eating habits [6]. Sometimes, everyday life situations affect not only adults but their children as well [7]. Parents have the primary role in promoting health and setting healthy dietary patterns and habits for their children [8]. Children may be led to consuming large portions of food through parental pressure even if they are not hungry [9,10]. Moreover, parents may provide non-healthy foods to reward their children’s behavior, and this may help them to adopt unhealthy dietary habits [11] and increase the risk of overweight/obesity.

Among other causal factors (such as genetic, behavioral, environmental, and socioeconomic) that have been identified in obesity, a very important one is the psychosocial aspect. Recently, studies have contained evidence that psychosocial stress is linked to obesity [12,13]. In particular, dietary habits such as increased food intake, food high in calories, or reduced consumption of low-calorie diets are linked to stress, and this may deleteriously impact weight and health status by increasing body adiposity [14,15,16]. Moreover, stress could determine children’s negative behaviors about their body and weight, leading to eating disorders such as binge eating disorder, bulimia or anorexia nervosa (through excessive worry about controlling their weight); the first one could be difficult to diagnose in childhood [17]. Such eating disorders may have consequences on children’s gain weight and obesity. Furthermore, children and adolescents face many demands related to their family, school, and conflicts with parents, siblings, friends, and classmates on a daily basis. All the above contribute to adolescents’ feelings of strain and stress [18]. Additionally, children’s food choices are influenced by TV and social networks, while video and online games dissociate them from outdoor and interactive activities [19].

There are various pathways through which stress is connected to obesity [20]. Stress can destabilize cognitive self-regulation processes that are responsible for food choices. Failures in inhibitory control can lead to stress-eating behaviors such as emotional eating, i.e., eating as a coping mechanism to soothe negative emotions, which has been blamed for weight gain [21]. Stress has also been found to be linked to reduced physical activity [22] and various adolescent health behaviors [23], increasing the risk for obesity. The recent evidence on how stress affects weight status in children is scarce and not well studied, and there is a gap of knowledge regarding this age group in our country. Thus, the purpose of the present study was to assess the association between stress and weight status through several characteristics in a representative sample of Greek children aged 10–12.

## 2. Materials and Methods

### 2.1. Design and Setting

This is an observational, school-based, cross-sectional study. From five cities of Greece, 47 schools were included in the research (i.e., 32 from the Athens metropolitan area, 5 schools from Heraklion, and 10 schools from the Peloponnese peninsula as described elsewhere [24]). Schools were randomly selected through a stratified random sampling procedure using a list of schools provided by the Greek Ministry of Education.

### 2.2. Sampling Procedure

Children from the 5th and 6th grades of primary school were eligible to participate in this research. The school years 2014–2015 and 2015–2016 were enrolled in the study, totaling 1728 participants between 10 and 12 years old. From school to school, the participation rate varied between 95% and 100%. For the purposes of this study, only children who completed the questions about the sources of stress were included. No difference was observed in the basic characteristics between children not included in the study’s sample and those included [24].

### 2.3. Measurements

A specially developed questionnaire containing various items was completed anonymously by the children. The questionnaire retrieved information about demographic characteristics (i.e., place of residence, sex, age), several other characteristics such as eating habits using the semi-quantitative Food Frequency Questionnaire [25], physical activity (using Lifestyle Questionnaire (PALQ) [26]), and stress management.

Given that the Mediterranean diet is largely followed by the Greek child population, the assessment of children’s adherence to the Mediterranean diet was conducted through use of the Mediterranean Diet Quality Index (KIDMED score) for children [27] as described elsewhere [28]. This is a standard, validated and widely used tool to evaluate adherence to the Mediterranean Diet for children and youths.

Children’s stress was assessed through self-report questions, which reflected five sources of stress: the stress of home life, the stress of teacher interaction, the stress of school performance, the stress of school/leisure conflict, and the stress of peer pressure, based on the Adolescent Stress Questionnaire (ASQ) [29] translated and evaluated in several countries, including Greece [30]. Only five questions from the ASQ were used as they are the only ones adequate for the studied age group to assess stress-related emotions. In addition, a single latent variable was created through multivariate correspondence analysis (MCA) on the aforementioned stress-related questions and it was then transformed through standardization in the more conceptual range of 0–100, with lower values indicating lower stress. The theoretical score’s range tertiles (0–100) were also calculated, denoting “low” (i.e., range ≤ 33/100), “moderate” (34–66/100), and “high” (≥67/100) stress levels.

Specially trained health scientists/investigators (i.e., dietitians, registered nurses, physicians) took the necessary anthropometric measurements of children (height and weight in cm and kg, respectively) using a tape measure and a scale (with skin-tight clothing, to minimize measurement errors) and performed a face-to-face interview with them, which lasted a maximum of 10–15 min. Children’s body mass index (BMI) was calculated as the ratio of kilograms/(height in cm)^2^ and the weight status was classified as either with normal weight or with overweight/obesity according to the International Obesity Task Force (IOTF) BMI cut-off criteria [31].

Furthermore, several parental sociodemographic characteristics, marital status, and educational level were recorded by the children’s parents. For marital status, two categories were used: nuclear families (i.e., a pair of married or unmarried adults and their dependent children) and single-parent families. For more information on the parental characteristics, the interested reader is referred to [32].

### 2.4. Statistical Power Analysis

The sample used in this study was adequate for the evaluation of minimum detectable standardized, two-sided differences of 20% on the prevalence of overweight/obesity, with 85% statistical power at a 5% significance level [24].

### 2.5. Bioethics

Before starting the study, approval was requested from the appropriate department of the Ministry of Education and Religious Affairs (code of approval F15/396/72005/C1 by the Institute of Educational Policy) and was carried out following the principles of the Declaration of Helsinki. The investigators informed all people who were involved about the aims and procedures of the research. The students participated in the study after the written consent of their parents.

### 2.6. Statistical Analysis

Mean and standard deviation (SD) were used for continuous variables, while absolute and relative frequencies (percentages) were used for categorical variables. The effect of stress on the children’s weight status was assessed through six nested logistic regression models by using BMI categories as the response variable. Specifically, the variables about children with normal weight vs. children with overweight/obesity were used as the dependent variable and the coded stress factor as the independent variable, adjusting for several confounders, i.e., age, sex, KIDMED score, type of family structure, type of residence, and parental educational level. Odds ratio (OR) and 95% confidence intervals (CI) were used to present all logistic regression results. MCA was used to reveal a single latent variable that assesses stress. This is a statistical technique for handling categorical data often encountered in biomedical research, among others [33]. The significance level was set at 5% and all statistical tests were two-sided. Analyses were conducted through statistical program Stata 14.0 (M. Psarros & Assoc., Sparti, Greece).

## 3. Results

### 3.1. Prevalence of Overweight/Obesity

In total, 1452 children, of whom 668 were boys, participated in the study. Their mean age was 11.24 ± 0.77 years old. The overall prevalence of overweight was 22.3% and of obesity was 4.7%. Sex-specific analysis revealed that 31.1% of boys and 23.5% of girls were categorized in the group of children with overweight/obesity (*p* = 0.001 for sex difference).

Children’s sociodemographic characteristics and lifestyle habits by body weight status are presented in Table 1. A significant association of body weight with sex (*p* = 0.001), engagement in physical activities (*p* = 0.002), parental educational level (*p* = 0.004 for mothers and *p* = 0.010 for fathers), and residence (*p* = 0.011) was revealed. Specifically, boys were more likely to have overweight/obesity compared to girls; children engaged in physical activities were less likely to have overweight/obesity compared to those who followed sedentary behaviors; children whose parents were higher educated were less likely to have overweight/obesity compared to those whose parents had basic/secondary education; and children from Athens were less likely to have overweight/obesity compared to children from other areas of Greece. No significant differences were observed between children’s eating habits and overweight/obesity status. Moreover, children with overweight/obesity had significantly higher adherence to the Mediterranean diet than children with normal weight.

### 3.2. Distribution of Stress Score among Children

The mean value of the stress score variable was 58.68/100 ± 27.48; sex-specific analysis showed that the mean stress score value was 60.38/100 ± 27.81 for boys and 57.23/100 ± 27.13 for girls (*p* = 0.029). Further analysis by the source of stress revealed that stress related to school performance and stress related to school activities/leisure conflict was more likely to be present among girls than boys (*p* < 0.001 and *p* = 0.029), respectively. No difference was found regarding the other three sources of stress.

### 3.3. Children’s Weight Status and Its Association with Stress

Children with overweight/obesity had significantly higher mean stress score than children with normal weight (62.42 vs. 57.30, *p* = 0.002). In Table 2, the crude associations between the five sources of stress studied here and children’s weight status are presented. Children reporting stress due to teacher interaction were 44.1% more likely to have overweight/obesity compared to those who were not stressed (OR (95% CI): 1.44 (1.14, 1.89), *p* = 0.002). Similarly, children reporting stress related to school performance were 32.2% more likely to have overweight/obesity compared to those who were not stressed (OR (95% CI): 1.32 (1.04, 1.67), *p* = 0.020). Children showing stress related to school/leisure conflict were 28.9% more likely to have overweight/obesity compared to those who were not stressed (OR (95% CI): 1.29 (1.02, 1.64), *p* = 0.036). Sex-specific analysis revealed that girls reporting stress related to teacher interaction were 62.6% more likely to have overweight/obesity compared to those who were not stressed (OR (95% CI): 1.44 (1.16, 2.29), *p* = 0.005).

### 3.4. Association among Children’s Sociodemographic Characteristics, Lifestyle Habits, and Stress

Children from single-parent families had a higher mean stress score compared to those of nuclear families (61.09 vs. 54.67, *p* = 0.021), as shown in Table 2. Moreover, children from nuclear families who were stressed by their teachers were 36.4% more likely to have overweight/obesity than those who were not stressed (OR (95% CI): 1.36 (1.00, 1.86), *p* = 0.048), whereas no significant association was found for children from single-parent families (OR (95% CI): 0.80 (0.33, 1.95), *p* = 0.620).

In Table 3, children’s sociodemographic characteristics and lifestyle habits by stress level are presented. An association between stress and children’s sex (*p* = 0.010), place of residence (*p* < 0.001), and family marital status (*p* = 0.015) was revealed. Specifically, the exploratory analysis revealed that children from single-parent families were more stressed compared to children from nuclear families; children living in urban or rural cities were also more stressed compared to children living in the Athens metropolitan area.

### 3.5. Association between Stress Factor and Children’s Weight Status

**Figure 1:**
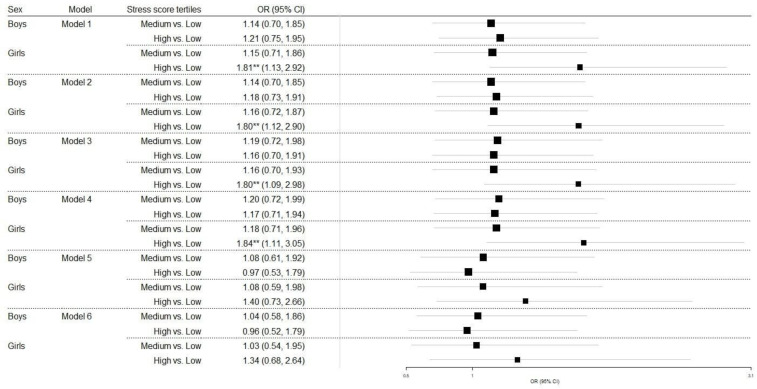
The association between stress and children’s weight status category (overweight/obese vs. normal), adjusted by sex. OR, odds ratio; 95% CI, 95% confidence interval; ** *p* < 0.05;.

However, residual confounding may exist due to the observational nature of the present study. In Table 4, the results from nested logistic regression models evaluating the association between stress and children’s weight status, after taking into consideration various children’s characteristics, are presented. In total, six models were estimated. As can be drawn from model 1, children with a high stress score were 51.7% more likely to have overweight/obesity compared to children with low-stress score (OR (95% CI): 1.52 (1.09–2.12), *p* < 0.05). No such association was revealed when comparing children with a moderate stress score to those with a low stress score. This association between stress score and weight status remained significant irrespective of sex, age, physical activity level of children, and KIDMED score (models 2, 3, and 4). However, when the parental educational level was taken into account (model 5), it was observed that stress score was not associated with children’s weight status, irrespective of all other factors included in model 5. This was also the case when the family structure and the place of residence were included in the final model (model 6). The association between stress and children’s weight status by sex is presented in Figure 1. A positive association was found only in girls (models 1 to 4).

## 4. Discussion

The present observational study investigates the effect of stress on the weight status of children aged 10 to 12 years living in Greece, after adjustment for several demographic and sociodemographic characteristics. More than half of the children appeared to have a medium or high level of stress, which is associated with the school environment. Various sociodemographic characteristics, as well as daily habits, were found to be significantly related to the weight status and stress of children. However, the most important finding is that school-related stress is positively associated with obesity in children.

The pathophysiological relationship between stress and obesity is indicated by literature evidence through several biological and behavioral pathways [34,35,36,37,38]. Indirectly, stress is also considered to determine health outcomes by changing a person’s activity with particular behaviors. For example, children’s eating habits may be influenced by a stressor that may finally lead to abnormal health behaviors, such as harmful dietary habits [39]. Children may adopt behavioral habits in response to exposure to stress, which are adaptive in the short term but harmful in the long term, such as eating comfort foods to reduce stress [12]. A cross-sectional study of 4320 school-aged children showed that higher levels of self-reported stress were associated with obesogenic eating behaviors (low consumption of fruits and vegetables, high consumption of high-fat foods, snacks, etc.) [40]. Emotional eating is a mechanism that many people use to cope with stressful events [41]. Moreover, behaviors such as abnormal eating may lead to eating disorders as a consequence, which may also increase the risk of obesity. However, sometimes the association between eating disorders and obesity appears to be bidirectional [42]. Furthermore, chronic family-level stress can also affect children’s levels of physical activity and obesity risk. As revealed by a cross-sectional study including 110 parent–child pairs, high levels of parenting stress were associated with less physical activity and fewer limits on time spent watching television among preschool-aged children [43].

Our analysis showed that school-related stress is positively associated with obesity in children. This is in line with the results of previous studies which revealed that school stress was significantly related to body fat [44,45]. School-related stress was more likely to be present among girls than boys. This result is consistent with that of a study on 545 Swedish students aged 14–16 [46]. This is rational as girls are usually more diligent and have a higher level of engagement at school [47] and perhaps this stresses them more than boys. Multivariate analysis revealed a constant positive association between stress and weight status when children’s characteristics (age, sex, physical activity, and adherence to the Mediterranean dietary pattern) were taken into account. This association ceased to be significant, when the sociodemographic characteristics of the parents were taken into account. It seems that the educational level of parents plays a crucial role both in terms of the weight status of children, through dietary choices, and the management of children’s stress. Low parental educational level is a high-risk factor for childhood obesity, possibly due to different cultural and social standards between parents of higher education and parents of lower educational levels [48]. A recent study of 2998 children aged 3–18 examining the relationship between parental SES and health parameters in German children and adolescents found an association between higher SES, lower BMI and healthier nutrition [49].

According to stress theory, changes in family structure often induce stress in a child’s life by reducing healthy eating, exercise, parental supervision, sleep routines, and emotional support, impacting their BMI and weight [50,51,52]. Compared to single-parent families, children of a younger age than our sample in nuclear households may have lower BMIs due to reduced stress levels and better emotional support and self-esteem [52]. Single parents are more likely to experience a lack of quality time with their children, due to work, and as a result, they may pay less attention to their children’s dietary choices, meal preparation, and mealtimes, allowing them to eat high-energy foods [51,53]. Furthermore, single parents may have less time to play with their children or to encourage them to engage in physical activities [51]. Due to a lack of time and resources, parents might adopt more convenient routines for their children, such as turning on the TV and allowing them to play video games, which could result in higher obesity rates [54]. Nowadays, this lifestyle is still followed. Moreover, concerning school, pupils at upper-social status schools might exhibit more unfavorable attitudes regarding obesity, probably either as a consequence of parental role modeling, which is usually characterized by dieting and healthy weight control practices, or even because of their commonly unrealistic body image standards, based on social media sites and platforms [48].

An impressive result of our study is that children do not consider their relationship with peers a source of stress. Usually, children with excess weight experience obesity stigma and a pressure to conform to another image of their body, and this perhaps increases stress in relationships with their classmates [55]. However, there is also the opposite view that peers motivate children with overweight/obesity to be more physically active [56].

It seems that children’s stress derives mainly from the school environment. It is generally accepted that school-based stress has an impact on social life, which is particularly demanding and competitive, and children often can hardly adapt to the school environment or cope with school demands and negative social experiences. Health education, including nutrition and exercise education, may be the most significant step against obesity. It could assist young people in improving their health status and making reasonable decisions about their personal health and psychosocial well-being by constructing values and beliefs that promote healthy lifestyles (e.g., participation in extracurricular activities). In order for such a campaign to have positive outcomes, school health education needs to collaboratively include parents or caregivers, teachers, and school counsellors who will work together in order to support this school action.

Given the cross-sectional design of our study, several limitations should be considered when interpreting the reported results. Mainly due to the observational study type, no temporal relationship and, hence, causal inferences can be made. Based also on the fact that contextual variables were used in the models, bias due to ecological fallacy may exist, and therefore, an association observed at the population level may not apply at the individual level. Moreover, the sample originated only from a small proportion of Greek areas. Thus, the generalizability of the findings is limited to the specific population of Greek children aged 10–12 years old. However, the representativeness of the study could be considered high given that the participating areas have similar characteristics with all other Greek areas, the study sample was large and a stratified random sampling scheme at the school level was used. Only five questions of the ASQ items were used here, as these were the only ones that fit for the studied age group; however, this short version has not been validated. Another limitation of the present study could be reporting bias during the completion of the questionnaire by the children in the school setting. However, to reduce this type of bias and increase the validity of the given responses, trained investigators were present throughout the whole procedure of completing the questionnaire in schools to address any potential misconceptions.

## 5. Conclusions

Our study supports that stress and, more specifically, school-based stress has a significant role in the weight status of children aged 10 to 12 years old. Parents’ educational level plays a significant role in both children’s weight status and stress. Children living in a nuclear family seem to be less stressed compared to those from single-parent families. Finally, additional research is required to further evaluate the effect of stress on children’s weight status and the role of the family environment.

## Figures and Tables

**Table 1:** Children’s sociodemographic characteristics and lifestyle habits, by body weight status.

Children’s Characteristics	Overall (*n* = 1452)	Normal Weight ** (*n* = 1060)	Overweight and Obese ** (*n* = 392)	*p* *
Age	11.24 (0.77)	11.26 (0.78)	11.17 (0.76)	0.060
Sex				
Boys	668 (46.0%)	460 (43.4%)	208 (53.1%)	0.001
Girls	784 (54.0%)	600 (56.6%)	184 (46.9%)	
Physical activity (yes)	1073 (79.1%)	802 (81.2%)	271 (73.6%)	0.002
KIDMED score ^†^ (−4 to 12)	4.65 (2.28)	4.74 (2.27)	4.43 (2.29)	0.023
Mother’s educational level				
Basic/secondary	526 (53.7%)	376 (51.1%)	150 (61.7%)	0.004
Higher	453 (46.3%)	360 (48.9%)	93 (38.3%)	
Father’s educational level				
Basic/secondary	575 (59.0%)	415 (56.7%)	160 (66.1%)	0.010
Higher	399 (41.0%)	317 (43.3%)	82 (33.9%)	
Residence				
Athens	970 (68.7%)	728 (70.6%)	242 (63.5%)	0.011
Other	442 (31.3%)	303 (29.4%)	139 (36.5%)	
Marital Status				
Married	873 (89.2%)	657 (89.4%)	216 (88.5%)	0.707
Non-married	106 (10.8%)	78 (10.6%)	28 (11.5%)	
PC (hours/weekday)				
≤3	1326 (91.3%)	967 (97.1%)	359 (95.7%)	0.210
>3	45 (3.1%)	29 (2.9%)	16 (4.3%)	
TV (hours/weekday)				
≤3	1313 (90.4%)	970 (95.2%)	343 (91.7%)	0.013
>3	80 (5.5%)	49 (4.8%)	31 (8.3%)	
PC games (hours/weekday)				
≤3	1298 (89.4%)	952 (96.4%)	346 (93.8%)	0.037
>3	59 (4.1%)	36 (3.6%)	23 (6.2%)	

SD = Standard deviation; IOTF = International obesity task force; BMI = Body mass index; PC = personal computer, TV = television. Children’s eating habits are presented as mean (SD) and categorical variables as frequencies (%), * Level of significance set at *p* < 0.05; tested via independent samples *t*-test for eating habits, and chi-square test for all other categorical variables ** Weight status is defined based on BMI cut-offs for adults and on IOTF cut-off criteria for children. ^†^ KIDMED score is presented as mean (SD).

**Table 2:** The association between sources of children’s stress and weight status.

		Normal Weight ** (*n* = 1060)	Overweight and Obese ** (*n* = 396)	*p*	OR (95% CI)
Home life/family related stress	Yes	372 (35.1%)	132 (33.7%)	0.614	1.06 (0.84, 1.36)
	No	688 (64.9%)	260 (66.3%)		
Stress related to teachers’ interaction/requirements	Yes	503 (47.5%)	151 (38.5%)	0.002	1.44 (1.14, 1.89)
	No	557 (52.5%)	241 (61.5%)		
School performance related stress	Yes	666 (62.8%)	220 (56.1%)	0.020	1.32 (1.04, 1.67)
	No	394 (37.2%)	172 (43.9%)		
Stress related with school activities/leisure conflict	Yes	468 (44.2%)	149 (38.0%)	0.036	1.29 (1.02, 1.64)
	No	592 (55.8%)	243 (62.0%)		
Peer pressure/classmates related stress	Yes	160 (15.1%)	56 (14.3%)	0.701	1.07 (0.77, 1.49)
	No	900 (84.9%)	336 (85.7%)		

OR, odds ratio, adjusted for …; 95% CI, 95% confidence interval; ** *p* < 0.05; * *p* < 0.10.

**Table 3:** Children’s sociodemographic characteristics and lifestyle habits, by stress factor.

Stress Score Tertile				
Children’s Characteristics	Low (*n* = 269)	Medium (*n* = 605)	High (*n* = 578)	*p* *
Age	11.28 (0.78)	11.29 (0.78)	11.17 (0.76)	0.016
Sex				
Boys	113 (42.0%)	264 (43.6%)	291 (50.3%)	0.010
Girls	156 (58.0%)	341 (56.4%)	287 (49.7%)	
Physical activity (yes)	201 (81.0%)	463 (81.1%)	409 (76.2%)	0.059
KIDMED score (−4 to 12)	4.48 (2.26)	4.60 (2.23)	4.78 (2.32)	0.165
Mother’s educational level				
Basic/secondary	109 (52.9%)	249 (54.0%)	168 (53.8%)	0.855
Higher	97 (47.1%)	212 (46.0%)	144 (46.2%)	
Father’s educational level				
Basic/secondary	120 (59.7%)	274 (60.1%)	181 (57.1%)	0.498
Higher	81 (40.3%)	182 (39.9%)	136 (42.9%)	
Residence				
Athens	208 (79.7%)	461 (78.8%)	301 (53.2%)	<0.001
Other	53 (20.3%)	124 (21.2%)	265 (46.8%)	
Family marital status				
Married	187 (91.7%)	417 (90.7%)	269 (85.4%)	0.015
Divorced/separated	17 (8.3%)	43 (9.3%)	46 (14.6%)	
PC (hours/weekday)				
≤3	239 (93.0%)	555 (95.4%)	519 (93.7%)	0.993
>3	18 (7.0%)	27 (4.6%)	35 (6.3%)	
TV (hours/weekday)				
≤3	248 (96.1%)	546 (96.3%)	532 (97.4%)	0.262
>3	10 (3.9%)	21 (3.7%)	14 (2.6%)	
PC games (hours/weekday)				
≤3	244 (95.3%)	551 (96.3%)	503 (95.1%)	0.699
>3	12 (4.7%)	21 (3.7%)	26 (4.9%)	

Children’s eating habits are presented as mean (SD) and categorical variables as frequencies (%), * Level of significance set at *p* < 0.05; tested via independent samples t-test for eating habits, and chi-square test for all other categorical variables. SD = Standard deviation.

**Table 4:** Results from nested logistic regression models (OR, 95% CI) that evaluated the association between stress and children’s weight status category (overweight/obese vs. normal), adjusted for several characteristics.

	Model 1	Model 2	Model 3	Model 4	Model 5	Model 6
Stress Score Tertile						
Low	1.00	1.00	1.00	1.00	1.00	1.00
Medium	1.15 (0.82; 1.62)	1.15 (0.82; 1.62)	1.18 (0.83; 1.68)	1.19 (0.84; 1.70)	1.08 (0.72; 1.64)	1.03 (0.67; 1.57)
High	1.51 ** (1.09; 2.12)	1.46 ** (1.04; 2.04)	1.44 ** (1.02; 2.06)	1.47 ** (1.03; 2.10)	1.16 (0.75; 1.81)	1.11 (0.70; 1.74)
Sex (boys vs. girls)	-	1.44 ** (1.14; 1.82)	1.45 ** (1.14; 1.85)	1.45 ** (1.14; 1.85)	1.70 *** (1.25; 2.31)	1.81 *** (1.31; 2.49)
Age (per 1 year increase)	-	0.88 * (0.76; 1.02)	0.84 ** (0.72; 0.98)	0.83 ** (0.71; 0.97)	0.80 * (0.66; 0.98)	0.82 * (0.67; 0.99)
Physical activity (yes vs. no)	-	-	0.62 ** (0.47; 0.83)	0.64 ** (0.48; 0.85)	0.63 ** (0.44; 0.92)	0.61 ** (0.41; 0.88)
KIDMED score (per 1 unit increase)				0.95 ** (0.90; 0.99)	0.96 (0.90; 1.03)	0.95 (0.89; 1.02)
Mother’s educational level (higher vs. basic/secondary)	-	-	-	-	0.72 (0.51; 1.02)	0.73 (0.51; 1.05)
Father’s educational level (higher vs. basic/secondary)	-	-	-	-	0.78 (0.55; 1.11)	0.72 (0.51; 1.05
Family structure (nuclear vs. single-parent)	-	-	-	-	-	1.08 (0.64; 1.84)
Residence (Athens vs. other)	-	-	-	-	-	0.86 (0.58; 1.28)

OR, odds ratio; 95% CI, 95% confidence interval; *** *p* < 0.001; ** *p* < 0.05.

## Data Availability

The data presented in this study are available on request from the corresponding author. The data are not publicly available due to privacy and ethical reasons.
